# GPAD: a natural language processing-based application to extract the gene-disease association discovery information from OMIM

**DOI:** 10.1186/s12859-024-05693-x

**Published:** 2024-02-27

**Authors:** K. M. Tahsin Hassan Rahit, Vladimir Avramovic, Jessica X. Chong, Maja Tarailo-Graovac

**Affiliations:** 1 Departments of Biochemistry, Molecular Biology and Medical Genetics, Cumming School of Medicine, University of Calgary, Calgary, AB T2N 4N1 Canada; 2 Alberta Children’s Hospital Research Institute, University of Calgary, Calgary, AB T2N 4N1 Canada; 3 Division of Genetic Medicine, Department of Pediatrics, University of Washington, Seattle, WA 98195 USA; 4 Brotman-Baty Institute, Seattle, WA 98195 USA

**Keywords:** Mendelian disorder, NLP, Gene-disease relationship, Gene discovery, Rare disease gene, Trends in gene discovery

## Abstract

**Background:**

Thousands of genes have been associated with different Mendelian conditions. One of the valuable sources to track these gene-disease associations (GDAs) is the Online Mendelian Inheritance in Man (OMIM) database. However, most of the information in OMIM is textual, and heterogeneous (e.g. summarized by different experts), which complicates automated reading and understanding of the data. Here, we used Natural Language Processing (NLP) to make a tool (Gene-Phenotype Association Discovery (GPAD)) that could syntactically process OMIM text and extract the data of interest.

**Results:**

GPAD applies a series of language-based techniques to the text obtained from OMIM API to extract GDA discovery-related information. GPAD can inform when a particular gene was associated with a specific phenotype, as well as the type of validation—whether through model organisms or cohort-based patient-matching approaches—for such an association. GPAD extracted data was validated with published reports and was compared with large language model. Utilizing GPAD's extracted data, we analysed trends in GDA discoveries, noting a significant increase in their rate after the introduction of exome sequencing, rising from an average of about 150–250 discoveries each year. Contrary to hopes of resolving most GDAs for Mendelian disorders by now, our data indicate a substantial decline in discovery rates over the past five years (2017–2022). This decline appears to be linked to the increasing necessity for larger cohorts to substantiate GDAs. The rising use of zebrafish and *Drosophila* as model organisms in providing evidential support for GDAs is also observed.

**Conclusions:**

GPAD’s real-time analyzing capacity offers an up-to-date view of GDA discovery and could help in planning and managing the research strategies. In future, this solution can be extended or modified to capture other information in OMIM and scientific literature.

**Supplementary Information:**

The online version contains supplementary material available at 10.1186/s12859-024-05693-x.

## Background

For decades, researchers have been discovering genes underlying Mendelian Disorders (MD)s and associating them with the phenotype. In 1983, for the first time, researchers were able to map Huntington's disease gene to chromosome 4 [1]. At that time, they used DNA polymorphisms and positional cloning to map the gene location. Later, the discovery and associating genes with a phenotype significantly improved with the advancement in genotyping technology and subsequently, the emergence of sequencing technology [2–4]. As a result, new insights regarding gene-phenotype associations have emerged in recent times [5, 6]. Concurrently, the progressive development of bioinformatics tools, methods, and databases helped significantly improve the discovery rate of GDAs [7–10].

OMIM was started as a catalogue of MDs and their traits [11, 12]. Initially released in the form of books, in 1987, it was later published as an online catalogue. Later, in 1995, it was made available to the world via world-wide-web by the National Center for Biotechnology Information (NCBI) of the United States. Over the last 60 years, OMIM has become one of the leading databases for MDs, tracking the GDAs from the very beginning of genetic association. It is a manually curated and continuously adapting resource that provides a synopsis of Mendelian phenotypes and associated genes.

**Fig. 1:**
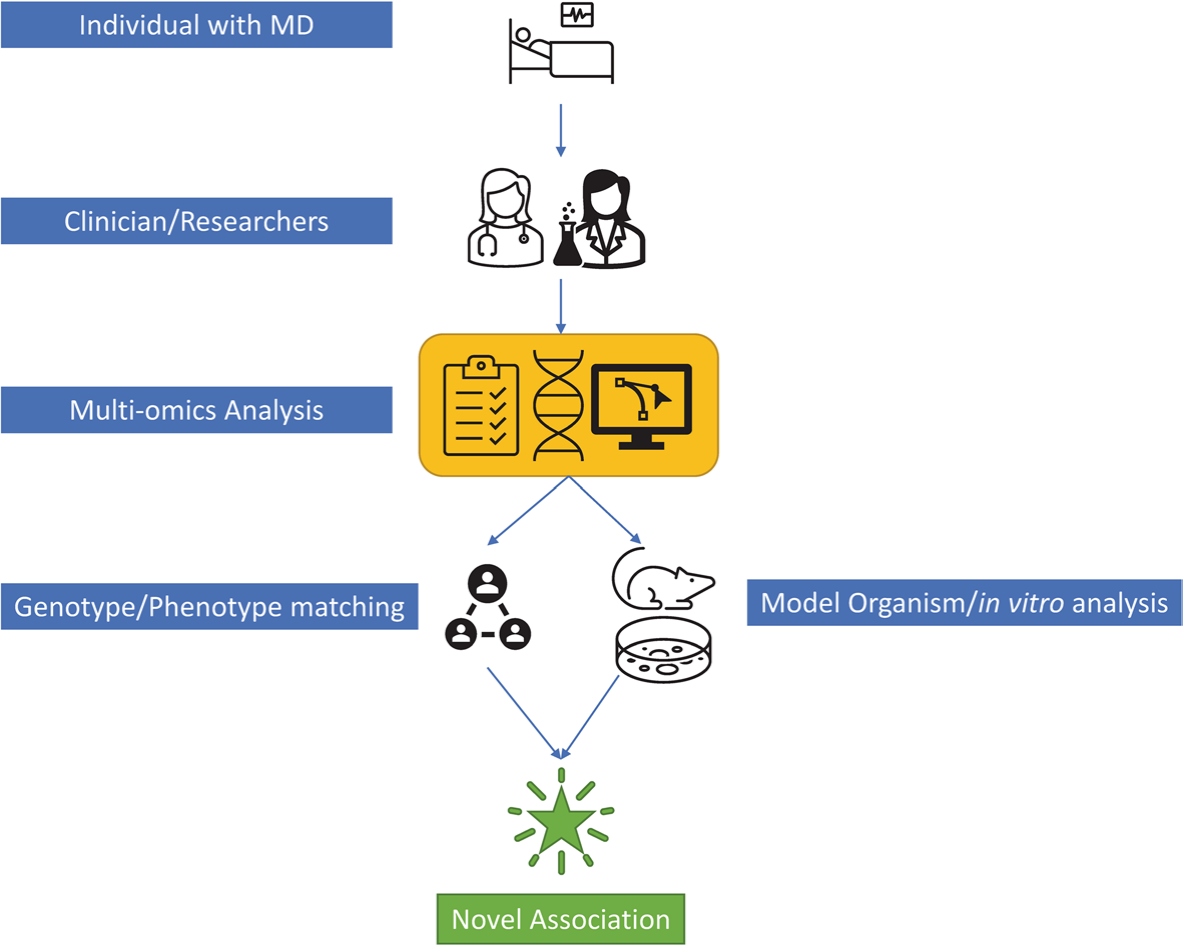
Process of novel GDA discovery. This Fig. illustrates the approach clinicians and researchers adopt when encountering an individual with a condition of unknown genetic origin. The process begins with a comprehensive multi-omics analysis, combined with the individual's health records, to pinpoint potential genetic causes. Subsequent genotype or phenotype-based matching efforts are employed to identify similar cases, facilitating the narrowing down of possible gene-disease associations. These associations are then typically validated through model organism studies and in vitro genetic experiments, leading to the establishment of a novel gene-disease association

While OMIM provides much valuable information on MDs, most of it is textual. For example, there are multiple commonly-used approaches to provide supporting evidence for new GDAs—(1) identifying dominant/recessive allele for a gene in multiple unrelated individuals with similar phenotype (e.g. using Matchmaker Exchange [13, 14]), (2) using model organisms, (3) performing in vitro functional studies, and (4) mapping the MD to a specific locus. Metadata about when a GDA was made and which methods [15–17], technologies, or experimental approaches [16, 18] were used to confirm the GDA (Fig. 1), are often described in text in OMIM but are not stored as separate fields that could be queried.

In addition, since it is a manually curated database, different human experts have written its texts over the years. As a result, the representation of information within the text is heterogeneous in nature making it difficult to mine using an automated program. To develop such automated program, semantic understanding of the text is important. Fortunately, advancements in Machine Learning (ML), especially in Natural Language Processing (NLP), could help significantly in this regard to obtain valuable insights from such texts [19, 20].

This study introduces Gene-Phenotype Association Discovery (GPAD), an application that allows its users to get key information regarding GDAs. In addition to when an association was made, it informs methodological supporting evidence that could be found on OMIM. The tool combines a series of NLP tasks and applies grammar-based pattern-miner to extract GDA discovery-related information from the text obtained from OMIM API. We have made the tool along with its web interface, available for anyone's use (https://github.com/MTG-Lab/gpad). Through GPAD, users can obtain the latest information as well as trends on GDA discovery. To the best of our knowledge, this is the first tool to allow real-time analysis of the rate of new GDAs discovery and explore methods used, which would facilitate developing a better understanding of the factors affecting GDA rates.

## Methods

GPAD’s implementation has three primary components: Firstly, a specialized algorithm for extracting information from the OMIM. Secondly, a series of validation phases to ensure accuracy of the extracted data. Lastly, a user-friendly, deployable web tool that facilitates access to the extracted data.

### Data extraction algorithm

#### OMIM API

OMIM provides API endpoints to access its data. We obtained an API key and used the API to access OMIM gene and phenotype descriptions. We will refer to these descriptions as “OMIM texts” to make it easy to explain. We made API calls to programmatically retrieve OMIM texts on December 16^th^, 2022. OMIM API allows 250 API requests per day per API key. Therefore, we tracked and analyzed OMIM text through the API over multiple days to reliably produce the graphs based on all GDAs.

#### Selection criteria for GDAs

OMIM has standards in-place to establish a GDA [11]. It uses specific prefixes (e.g. *#*, *, +, *%*) and symbols (e.g. brackets—[], braces—{}, question mark—?) and genotype-phenotype mapping key (denoted as integer number—1, 2, 3, 4) to denote the certainty and status of its entries (Additional file 1: Table S1) [21]. GPAD utilizes these markers to initially filter out associations that are yet not confirmed. After filtration, we identified 5236 confirmed GDAs (Table 1).

**Table 1:** Number of GDAs in each filtration step

Data description	Number of GDAs	Filter criteria
Total extracted	7484	
Keep only mapping key “3”	6313	Filter out Mapping Key not equal three (n = 1171)
Keep only confirmed association	5236	Filter out phenotypes marked with—“susceptibility”, “modifier”, [] or {} or ? (n = 1077)
GPAD identified publication evidence for	5198	
Cohort related information found for 0 (Same study as the association making study)	5043 (3554)	
GDA utilizing Animal model	3730	

**Fig. 2:**
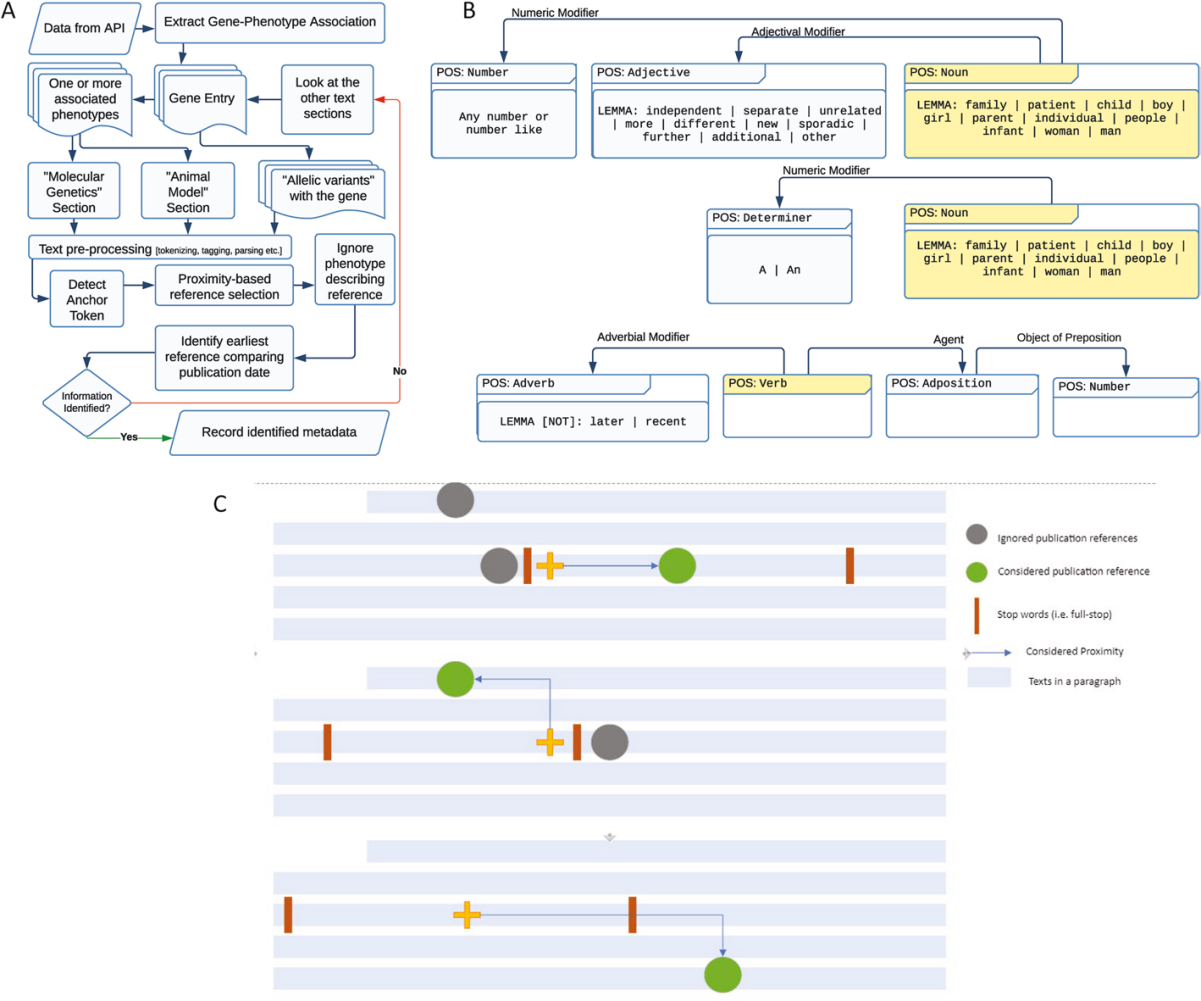
Method used in metadata extraction. **A** Schematic diagram of overall NLP workflow. **B** Three major grammatical rule-based dependency patterns. The first and second pattern extract cohort related information and the third one is used as an exclusion pattern to ignore phenotype only describing studies. Anchor token for each pattern is depicted in yellow. **C** Schematic diagram showing mechanism of proximity-based anchor token detection within a context of three different paragraphs. Here we depict three possible scenarios where the identified token (green circle) is present in the paragraph and how we use the relative distance from the reference anchor token (yellow cross) to identify the target token (circles). Stop words (orange bars) denote sentence boundaries

By computationally analyzing the paragraphs (Fig. 2A, Table 2) on the gene and associated phenotype entry page, we extract information that is not available as quarriable information. In particular, we look for three types of evidence in the text:

**Table 2:** Information GPAD extracts, prioritized source of each piece of metadata, and extraction specification

Information	Source paragraph from OMIM	Specification
Gene-phenotype association	Gene-Phenotype Relationships	Inheritance type is Mendelian Clear association has been established
When was the association first made?	“Molecular Genetics” of the phenotype entry page “Allelic Variants” on Gene entry page	Literature evidence that did not only report the patient but also made the association with the disease phenotype
Number of unrelated individuals in publication that established GDA	“Molecular Genetics” of the phenotype entry page “Allelic Variants” on Gene entry page	Contains any of the anchor following tags as noun POS: family, patient, child, boy, girl, parent, individual, people, infant, woman, man An adjective modifier that modifies the noun anchor tag and specify the patient as unrelated A numeric value (either in words or as number) that specify the number of patients Negate pattern: To ignore phenotype describing studies we look for a patten of Adverb-Verb-Adposition-Number
Model organism	“Model Organism” paragraph on phenotype entry page “Molecular Genetics” of the phenotype entry page “Allelic Variants” on Gene entry page	Look for names of model organisms as noun POS

(1) when the association was made; (2) the number of unrelated, affected individuals included in the study(ies) establishing the GDA; and (3) whether any model organism was used to provide supporting evidence for the association. Sequencing and analysis of unrelated individuals diagnosed with the same MD [22](i.e. phenotype-driven/phenotype-first gene discovery) and gene-based matchmaking followed by delineation of a shared MD [13, 14](i.e. genotype-driven/genotype-first gene discovery) have both been effective strategies for establishing GDA alongside model organism work. We scan for a list of 12 commonly used species/animals to identify studies where the new GDA was modelled using model organisms.

OMIM texts are curated by many individuals over the span of several decades, resulting in significant variability in the representation of information. To address this, we use a grammatical rule-based dependency pattern matcher (Fig. 2B) to pinpoint and extract specific data. In our approach, each word (or token) in the text undergoes parts-of-speech (POS) tagging and named entity recognition (NER). For executing various NLP tasks, such as tokenization, tagging, lemmatization and parsing, we utilized the SpaCy [23] Python library, capitalizing on its pre-trained model (*en_core_web_sm-3.7.0*), matcher APIs, and visualization tools. Subsequently, a grammatical dependency tag is attached to each token by SpaCy, creating what is termed as "dependency tree". An example of such a dependency diagram is presented in Fig. S1A, Additional file 1.

#### Pattern mining for cohort/model organism metadata

Within the workflow of GPAD, we resort to the text-pattern matcher at places where we need to identify particular words or phrases. These text patterns are a combination of regular expression, grammatical attributes, and phrasal structure. In this context, a "pattern" refers to a set of grammatical and relational dependencies among tokens that consistently point to the type of information we aim to extract. In each of these patterns, certain tokens serve as "anchor token"—the primary focus words that the rest of the pattern revolves around (Fig. 2B; anchor tokens are yellow color coded).

To extract information about the cohort describing study, we first identify the grammatical dependency pattern by analyzing its components (Fig. 2B). The development of the patterns starts with a rather simplified version of the pattern. Each token identified by this initial pattern is then scrutinized for its grammatical roles and relationships (Additional file 1: Fig. S1B–E). Based on this analysis, we introduce specific lemma (or root word) constraints (e.g., patient, family, unrelated etc.) and “exclusion pattern” (Fig. 2B). These strategies help in negating and filtering out noises (irrelevant information), enhancing its accuracy (as described in the Expert Validation section below by comparing the baseline from phase 1 vs final accuracy). Exclusion pattern is especially crucial in distinguishing studies- emphasizing those that identify causal variants rather than merely describing the disease.

#### Workflow for metadata extraction and publication evidence identification

Fig. 2A shows the schematic diagram of the GPAD workflow. Based on which question we are focusing on, we look at specific paragraphs of the OMIM gene entry and its associated phenotype entry page(s). Table 2 describes the source paragraphs and their specification for each type of metadata extraction. Here we use *CUL3* gene (MIM #603136) as an example. GPAD first extracts the gene-phenotype association from the “Gene-Phenotype Relationships” table (Additional file 1: Fig. S2). OMIM has two phenotypes (MIM #619239 and #614496) associated with *CUL3*. For the first GDA, that is—*CUL3* association with Neurodevelopmental disorder with or without autism or seizures (NEDAUS), we consider the source text section specified in Table 2.

Additional file 1: Fig. S3 shows how the GPAD workflow in Fig. 2A extracting GDA works for these particular pairs. To extract the earliest publication evidence (“When was the association first made?” in Table 2) for the GDAs, we first consider “Molecular Genetics” section in the phenotype entry page (as described in Table 2). For this part, we look for Anchor token 1—that is the gene name—*CUL3* (Additional file 1: Fig. S3).

Following the extraction of relevant tokens, we turn our attention to literature identification. Using the positional information of the anchor token as a starting point, we employ a “proximity-based” method to scan for publication details within the same sentence (Fig. 2C). It first looks for publication within the same sentence as anchor token, rejecting other references in the paragraph (e.g. 1st paragraph depict in Fig. 2C). If the search comes up empty, the algorithm scans preceding sentences in reverse order (e.g. 2nd paragraph in Fig. 2C), and if needed, sentences that follow (e.g. 3rd paragraph in Fig. 2C), up to the end of the paragraph. The goal is to locate the earliest manuscript that provides evidence of the GDA under consideration. In the case of *CUL3-*NEDAUS pair, the first citation is within the same sentence context of the anchor token 1 (Additional file 1: Fig. S3).

We then check if the selected publication is a phenotype describing study. We apply the third pattern from Fig. 2B to the paragraph and get all studies that report only the phenotype. If the publication selected from the previous step is one the phenotype/patient reporting study, we ignore the publication and move to the next citation. For *CUL3*-NEDAUS GDA, we do not have any phenotype describing studies in the set of five citations, thus Thiffault *et al*.’s study does not match with the phenotype describing pattern, GPAD selects this study as evidence for GDA (Additional file 1: Figs. S3, S4).

Next, to identify the cohort specific metadata, we apply the first two patterns specified in Fig. 2B. The pattern matches Anchor Token-2 from the paragraph (Additional file 1: Fig. S3) and applies the proximity-based reference publication selection processes described above with the same policy. Once, the earliest publication reference that is not a phenotype describing study (excluded using third pattern in Fig. 2B) is identified, we take the numeric modifier from the matched pattern to get the number of individuals that were studied.

Finally, for the model organisms, “Animal Model” section of the phenotype entry has the highest priority (as described in Table 2). The name of the model organism is used as focus word (Anchor Token-1, mice, in Additional file 1: Fig. S3). With this anchor token as reference, we calculate the proximity of the reference and select the closest and the earliest (Additional file 1: Fig. S4).

### Web tool

**Fig. 3:**
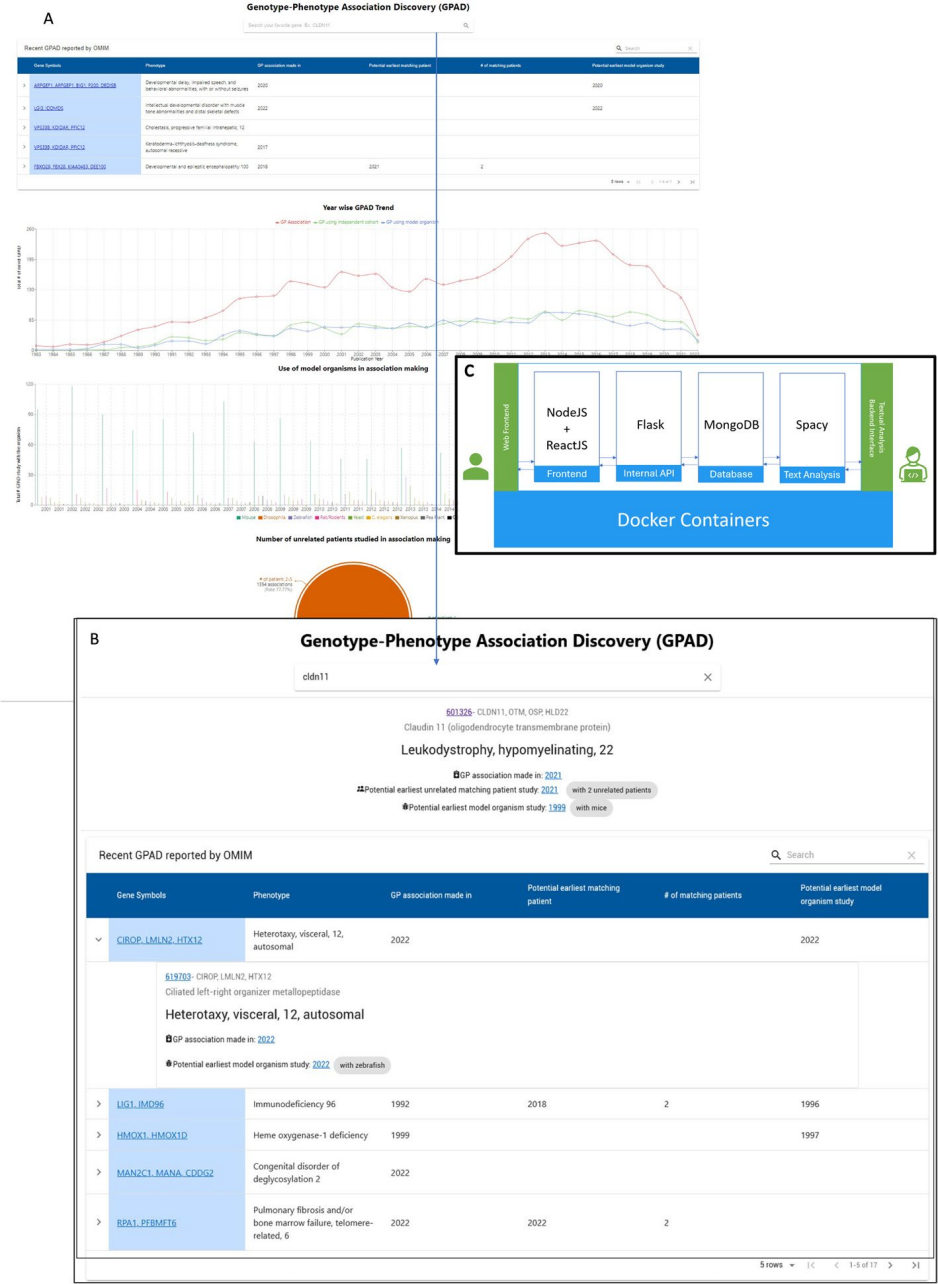
GPAD web tool. **A**, **B** Snapshot of GPAD web interface. **A** Snapshot of homepage with search option, latest OMIM reported GDAs, and latest trend graphs. **B** Snapshot of a search for a gene of interest by gene symbol (e.g. *CLDN11*). **C** Architecture of the web tool. All components have been containerized and shipped using Docker (Docker Desktop is a containerization app that lets user deploy, run, and ship stack of applications. https://www.docker.com.) to make it deployable on any system easily. Arrow denotes data and/or process communication. The web interface is developed with NodeJS (A JavaScript library used as an asynchronous event-driven runtime environment for frontend application. https://nodejs.org), React (JavaScript based React framework allows component-based frontend application development. https://react.dev) while Flask (A Python-based server-side framework. https://flask.palletsprojects.com) serves the backend API written in Python. We perform textual analysis with Spacy (Spacy library provides APIs for production level NLP tasks. https://spacy.io) library and the data are saved in a local MongoDB (a no-SQL database. https://www.mongodb.com) instance

The GPAD web tool gives users a comprehensive and detailed view of GDA discovery. We packaged the application with a graphical user interface (GUI) for easier exploration of GPAD data (Fig. 3A, B). The code base for GPAD is available at https://github.com/MTG-Lab/gpad. It requires OMIM API access to run the tool. Anyone interested in getting an overview can apply GPAD to gain the view at any time, as noted in OMIM. The complete application is built on top of *Docker* (https://www.docker.com) containerization system; making it deployable on any operating system with no additional software requirement other than the *Docker* itself. It uses the latest progressive web application (PWA) technology to conveniently browse both the web and as a mobile application. GPAD's data processing and analysis unit is developed with Python programming language with Flask Framework, and the visualization component is managed by NodeJS Framework (Fig. 3C). Installation instructions for the tool are provided in the *README.md* file that can be found in the GitHub repository.

After successful installation, the application can be viewed via a web browser. Figure 3A and B shows a snapshot of the homepage of GPAD. Figure 3B shows a snapshot of the search result view. The search option allows users to search by gene symbol. In the result box, all the associated phenotypes will be listed along with information about when the association was made and how it was made. The most recent associations that are added to OMIM are displayed in a table. Users can expand the row to view the details about a particular association. Furthermore, with the latest data, users can visualize all the trend graphs presented in this paper on its homepage as shown in Fig. 3A. It shows the yearly trend in novel GDA discovery, and the supporting evidence used in the GDA (matching patient and/or model organism). Separately, it reports the trend of the model organism utilization.

### Evaluation of GPAD’s performance

To ensure integrity and reliability, we employed both manual and automated validation of the extracted information (Additional file 1: Fig. S5).

#### Expert validation

In different phases, a total of 571 GDAs were manually checked to improve and validate GPAD’s performance. Extracted data was examined manually and independently by two people. They were tasked to identify the information by reading the text without prior knowledge of the text processing algorithm explained in the methods section.

In the first phase, 150 GDAs were chosen for manual validation. The genes for these GDAs were updated on OMIM between 2017 and 2022 and chosen randomly from the pool of genes that were updated during this period. We chose this timeframe to validate the most recent GDAs thoroughly as we did not have any recent data to automatically compare with (see Benchmarking Dataset below). During the manual validation, it was observed that GPAD missed a few infrequently-used model organisms such as—cattle, bull, chicken, dog, etc., which we then added to our list of species.

Notably, the expert validation revealed that the patients with specific phenotype were initially reported by an earlier study and noted as GDAs in many cases. However, these initially reported patients were only further studied and linked with a particular gene by a later study. We aimed to detect the study that made the GDA connection (the latter one). Still, our algorithm reported the earlier study (the study that only reported the patient phenotype but did not make the association). To solve this issue, we integrated another textual dependency pattern matcher to reliably identify the study by ignoring phenotype describing and/or patient reporting studies (Fig. 2B). After incorporating this mechanism into our algorithm, the detection accuracy improved, which was revealed by examining a new set of 100 GDAs.

We also compared dates of GDA discovery as assessed by GPAD and by scripts developed by Chong et al. [24]. Manual expert curation of the discrepancies where Chong et al. reports different dates compared to GPAD revealed that more than 50% was due to how OMIM describes the initial delineation of the MD, initial case report, or mapping study, followed by describing the publication discovering the GDA. A three-way comparison with the result from automated validation with benchmarking dataset (described below) shows that GPAD overcomes these limitations with its text-processing methods.

#### Benchmarking dataset

We also validated our NLP algorithm’s performance with previously published dataset. A recent study manually curated gene discovery metadata primarily from OMIM and other secondary sources such as PubMed, Wikipedia, and Google Scholar [25]. We utilized their data to validate our automated discovery. We do this by comparing the PubMed ID (PMID) [26] extracted by GPAD for each GDA with the PMID that was reported by Ehrhart *et al*. After performing automated validation, we have also manually reviewed all 281 discrepancies (Fig. S5, Additional file 1).

#### Comparison with large language model

The GDAs for which GPAD does not match with Ehrhart *et al.*’s report, we tasked Llama-2 to extract information for those. We used the model with 7 billion parameters with chatting capability (7B-Chat version). This model has a limit of context length of maximum 4096 tokens. We used high performance computing system with the parameters specified in Additional file 1: Table S2.

We have queried Llama-2 for 100 randomly selected GDAs out of 281 mismatched GDAs. Because of limited context size (4096 tokens) and to reduce the burden on Llama-2 of contextualizing the full entry page, we prompted Llama-2 with only the text from “Molecular Genetics” section. Additional file 1: Fig. S6 shows the template that we use for the prompt for each GDA. Once we get a structured reply from the model, we manually validate randomly selected 40 GDAs.

## Results

Leveraging the GPAD, we conducted a real-time extraction and systematic analysis of 5,236 GDAs catalogued within the OMIM database. The outcomes of this analysis are detailed as part of the results. To ascertain the robustness and efficacy of our methodology, we undertook a comparative evaluation against data and methods delineated in independent scholarly publications. All the results and analysis presented in the paper concern the data retrieved on 16 December 2022.

### GDA Trends Obtained Through GPAD

#### Overall GDA trend shows a recent decline in GDA discovery studies

**Fig. 4:**
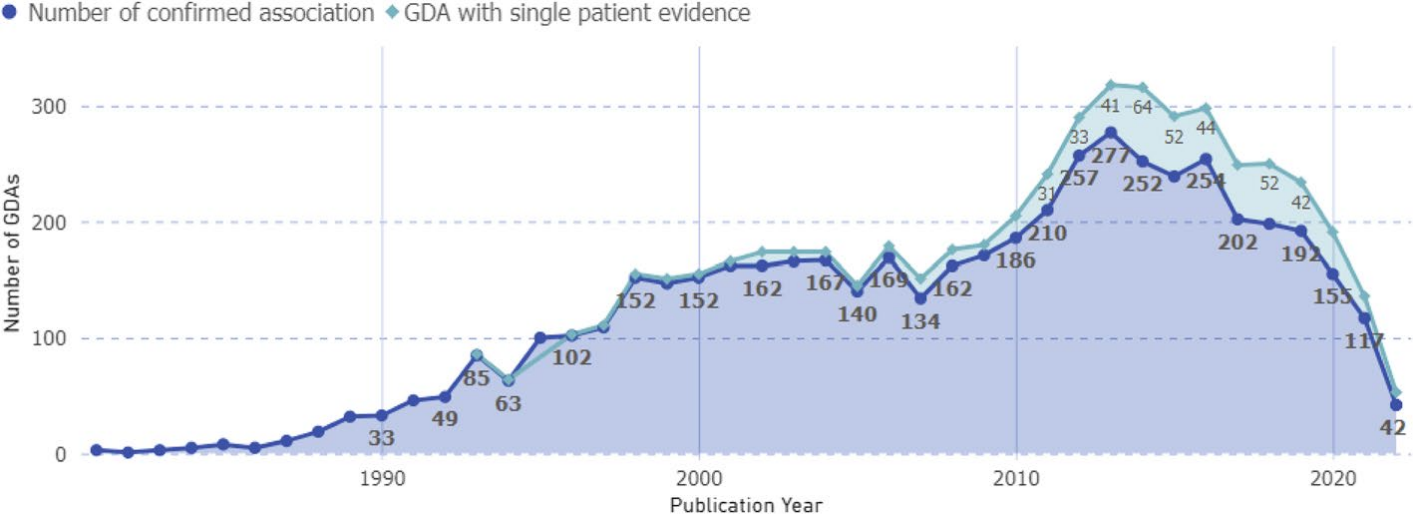
Trend analysis of GDA discoveries over time. Each dot represents the yearly count of identified GDAs. The green lined area represents GDAs currently categorized as 'provisional' in the OMIM database, identified based on single-patient evidence. Provisional GDAs, pending further verification via genotype–phenotype correlations, and laboratory studies, hold the potential to advance into the category of confirmed (blue line) associations

We confirm prior published analyses as well as present an up-to-date view of yearly trends in GDA discovery as shown in Fig. 4. The rate of GDA discovery increased through 2016 and has since declined. In 2013, the highest number of associations were made in a year. From 2011 to 2017, every year, at least 200 associations were made. This rapid increase can be explained by the improvements related to High Throughput Sequencing (HTS) technology and its integration into clinical and research assays [4, 27].

Compared to three decades back (1990–2000), the average number of GDA discoveries (88/year) has increased by 2.5 folds to 220 discoveries per year during 2010–2020 (Fig. 4). From the trend graph, the most active period was 2012–2016, and the number of GDAs made per year decreased after 2016. During this period, around 255 GDAs were made per year, or 300 GDAs if the single patient discoveries are included.

In contrast, the sharp decline of 2020 could be attributed, at least in part, to the Coronavirus Disease of 2019 (COVID-19) pandemic and the resulting crisis [28, 29]. During the COVID-19 pandemic, reduced access to healthcare, labs, and sequencing facilities, external responsibilities for Clinicians [30, 31] (responding to the COVID-19 emergency), changed priorities for researchers [32] (e.g. home-schooling and childcare), and increased research focus on COVID-19 clearly impacted rare disease studies and likely the ability for knowledge curators like OMIM to keep pace with the literature.

### Trends in discovery methodology

While there exist multiple approaches to provide supporting evidence for new GDAs, GPAD identifies two commonly used methods that OMIM provides as evidence—[1] identifying the same underlying gene in multiple individuals with similar phenotype and/or, [2] using model organisms. The evidence used in each GDA publication is not systematically documented in every OMIM entry, however where available, GPAD attempts to identify when OMIM does describe the use of multiple unrelated affected individuals (“cohorts”) and/or the use of model organisms.

**Fig. 5:**
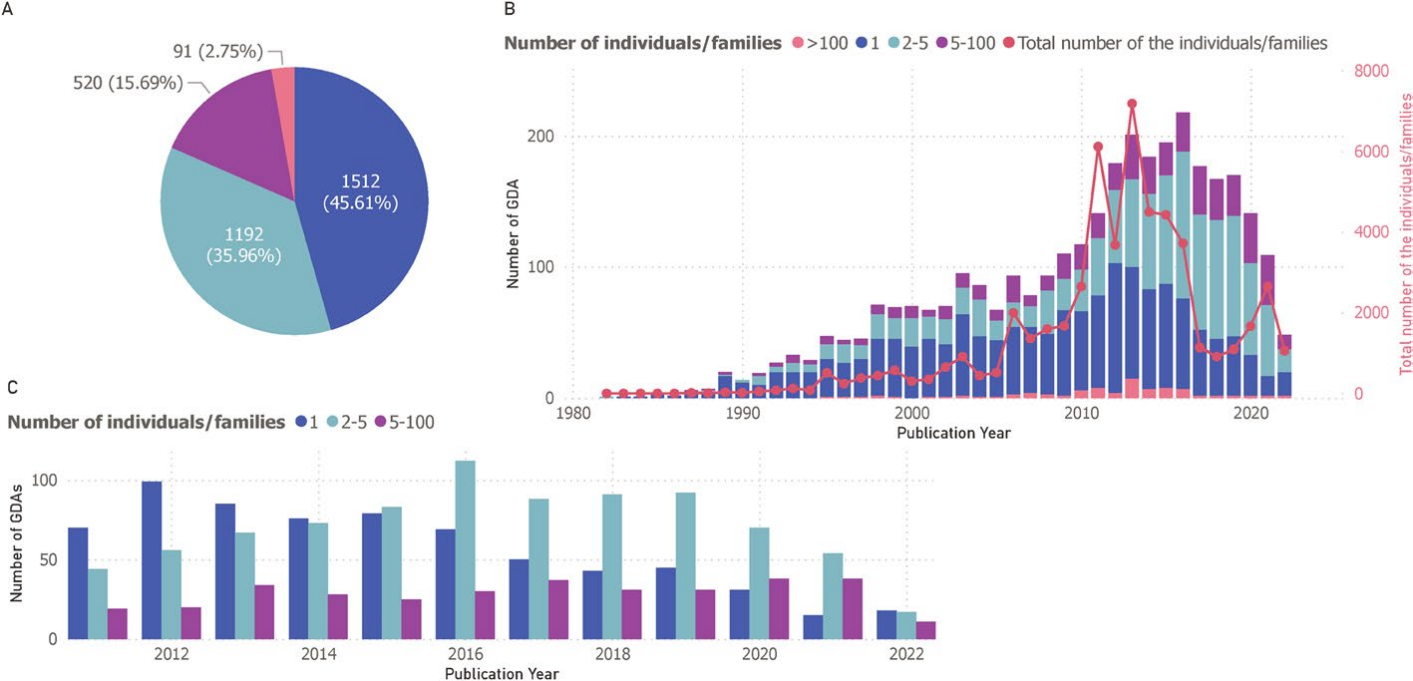
Number of individuals/families studied to make an association. **A** Displays a pie chart representing the count of unrelated individuals/families involved in initial GDA studies. **B** Illustrates the yearly trend (on the X-axis) in the number of individuals or families associated with GDA discoveries, with the left Y-axis detailing the annual total of GDAs and the right Y-axis (and accompanying red line) indicating the annual count of individuals or patients. **C** Provides a histogram focusing on GDAs identified with fewer than 100 individuals or families, offering a detailed view of studies involving smaller cohorts

GPAD extracted earliest cohort related information from the text for 96.31% [5] of the GDAs. As GPAD is capable of extracting publication information too, we compared earliest cohort study publication reference with the association making study. GPAD was able to identify the cohort information for 67.88% (3554) total GDAs. Fig. 5A shows the number of individuals studied to make the association. Expectedly, we observe that most associations (81.57%) were made by studying less than 5 unrelated patient cohorts.

The trend graph for cohort size (Fig. 5B) shows that in recent years the discovery was made possible with larger cohort size compared to before 2010. The total number of unrelated patients studied in a single year has decreased significantly in recent years (2017-onwards); also, it has become more common to report more than one patient after 2015.

**Fig. 6:**
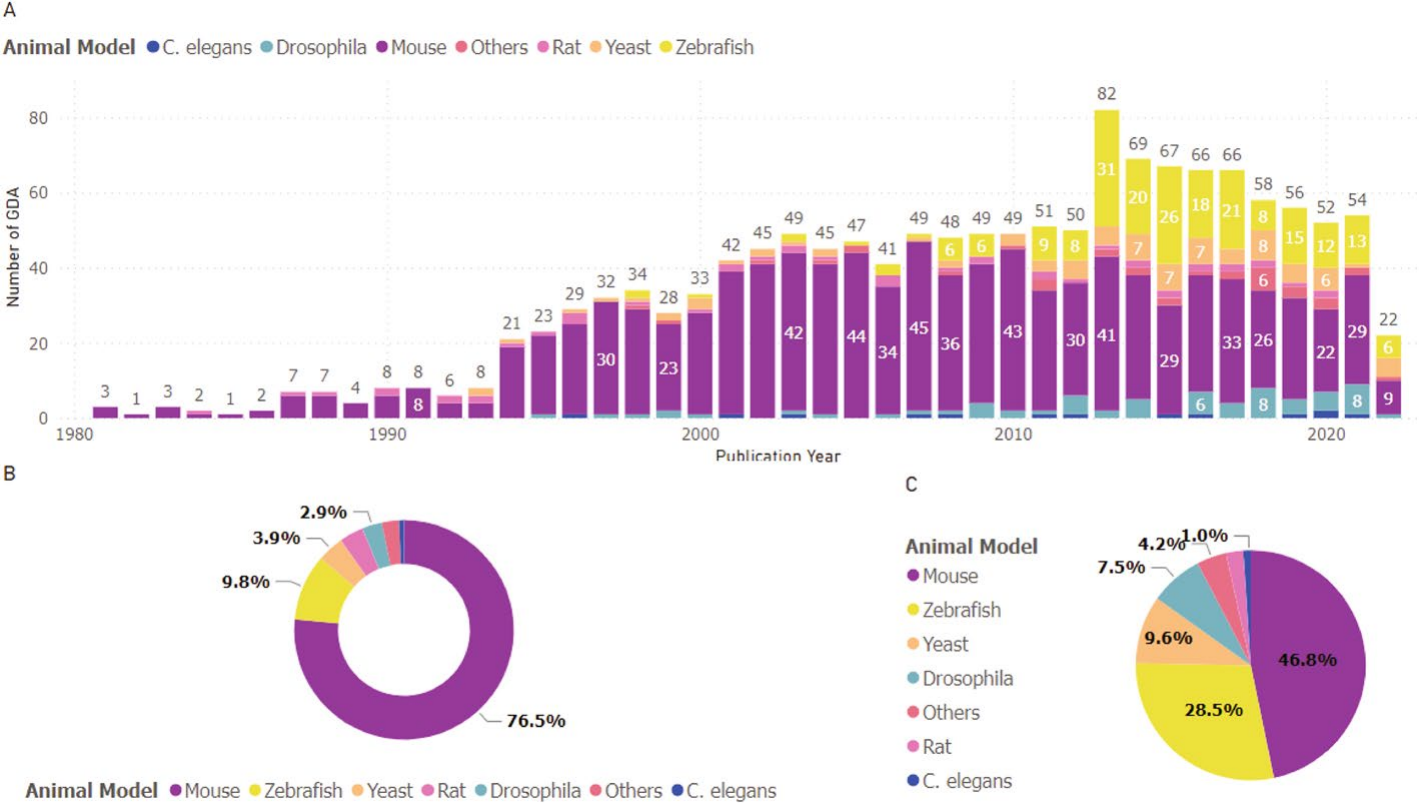
Model organism trends in association discovery. **A** Depicts the overall trend of GDAs reported with each bar indicating the animal model usage for a particular year. **B** Presents a donut chart demonstrating the predominance of mouse models in GDA studies historically. **C** Offers a pie chart focusing only on the last decade (2011–2021), revealing a diversification in model organism use

We further analyzed the studies for which GPAD has extracted single patient/family as a cohort and found that 70.10% [1060] of these studies were complemented by experiments with model organisms. For example, combined oxidative phosphorylation deficiency 10 (COXPD10; MIM#614702) is associated with defects in *MTO1* (MIM# 614667). The first report for this GDA was published in 2012 where they have found *MTO1* variant in two siblings [33]. The authors also performed *in vivo* experiments using yeast model organism. To provide a deeper look into the model organism study, we present a model organism utilization trend over the years in Fig. 6.

Compared to GDA discovery trend and cohort study trend, studies with model organism were comparatively stable as these do not show significant decrease (Fig. 6A). From 2011 until 2021 between 50 and 70 model organism studies were done with 2013 having a comparatively higher number (82). We have found that the mouse model has been the most popular over the years (Fig. 6B), most likely, due to the high availability of diverse phenotypes akin to human. In recent years (2013-onwards), the zebrafish has emerged as a prevalent alternative in approximately 28.5% association-discovery studies (Fig. 6C). Zebrafish offers efficient high-throughput screening techniques, and their progenies are easily maintainable compared to mouse [34, 35]. Furthermore, embryos of zebrafish develop outside of the mother allowing embryonic phenotyping. As a result, developmental disorders that affect embryonic development are easier to model. This same reason also applies to simpler model organisms such as worms, which are also being utilized in studying association discovery at an increasing rate [36, 37].

#### Performance analysis

We performed both manual expert validation and automated comparison with independently published dataset to evaluate the performance of GPAD as an automated data mining tool. In different phases, we have manually checked 571 GDAs in total. Initially, 150 associations were checked of which 66.67% (n=100) were correctly associated with the model organism described in OMIM as providing experimental evidence in the manuscript. The purpose of this evaluation was to identify potential improvements to GPAD. Later, we checked another set of 100 GDAs of which 48 (96%) GDA studies were identified correctly. For the cohort information, 72% (72) were accurate, 21% [21] were partially accurate, and the rest (7%) were inaccurate. The partially accurate GDAs often recognized the large cohort studies, among which only a few individuals were found to be carrying the variant responsible for the disorder. As for the model organism studies, 98% (98) were correctly identified.

**Fig. 7:**
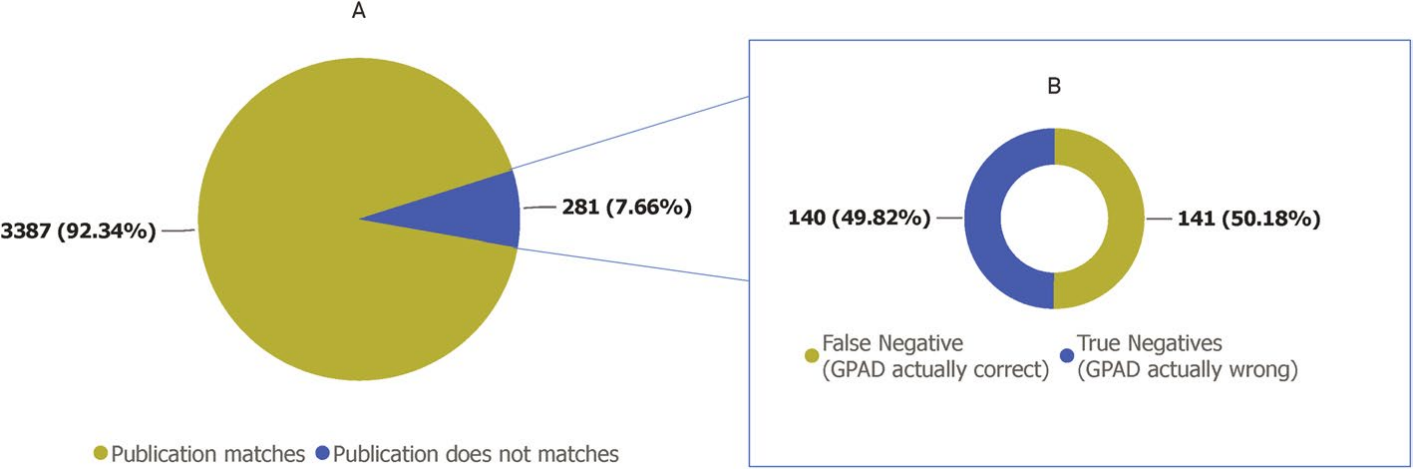
Comparative Performance Analysis of the GPAD. This Fig. shows the performance of GPAD by comparing its extracted PubMed IDs (PMIDs) against those identified in the study by Ehrhart et al. [25]. **A** Displays a pie chart illustrating the proportion of matched (green) and unmatched (blue) PMIDs between GPAD and the Ehrhart’s study. **B** Presents the results from a manual evaluation of 281 Gene-Disease Associations (GDAs) that were not automatically matched with the Ehrhart study (Blue part of the pie chart in **A**)

We used Ehrhart *et al*.’s dataset as benchmarking dataset for our automated evaluation [25]. This independently published data was collected and curated manually by the authors. Among 5236 GDAs identified by GPAD, 1568 associations were not present in benchmarking dataset, of which 58.77% (925) can be accounted for by GDA established after Ehrhart et al. finished their data curation (seemingly near the end of 2016/early 2017). Of the remaining 3668 GDAs, GPAD correctly identified 92.34% (3387) GDA publications and conflicted with Ehrhart *et al.* for 7.66% (281) (Fig. 7A).

Manual evaluation of these 281 discrepancies revealed that GPAD identified the correct publication for 141 (49.82% of the conflicts) (Fig. 7B). For example, Ehrhart *et al.*’s study identified Wynne Jones et al. [38] as the association-making study for Fletcher factor (prekallikrein) deficiency (OMIM ID: 612423) and *KLKB1* (MIM number: 229000). Whereas GPAD recognizes Lombardi et al. (2003) [39] for the association. This could be because of updated information on OMIM, as this record was last updated in 2022, and Ehrhart *et al.*’s report is based on 2015’s information.

We have found at least three cases where Ehrhart *et al.* reported “Exclusion Studies” as the association-making study. One such case is the association between Familial hypercholesterolemia-4 (FCHL4; OMIM#603813) and *LDLRAP1.* Sun *et al*. studied the possible link between the disease phenotype and gene in 1997 [40] but could not confirm any association. Thus, OMIM describes the 1997’s as an “exclusion study.” However, Ehrhart *et al*. report Sun *et al*.’s study as the association-making study. In this context, GPAD correctly identifies a study that was published two years later, in 2001, by Garcia *et al*. as the association-making study. A similar observation was made for Mitochondrial DNA depletion syndrome-7 (MTDPS7; OMIM#271245) and Fish-eye disease (FED; OMIM#136120).

Considering the 141 false negative GDAs, the overall accuracy increases to 96.16% (3528) (Fig. 7B). Our manual validation also shows similar accuracy of 96%. Of the 3.84% (140) GDAs for which GPAD incorrectly identified the association making publication, more than half of it is because of the lack of generalizability of GPAD’s algorithm to capture all the delineations. More specifically, the selection of reference publication based on the anchor token’s position in the text is mostly causing misidentification. Furthermore, for at least 10% of these 141 GDAs, multiple association-making efforts made the discovery possible, or multiple studies independently reported the GDAs at the same time. One example is variants in the *CCDC65* (MIM number: 611088), known to cause autosomal recessive ciliary dyskinesia (MIM number: 615504). In 2013, two independent studies simultaneously discovered this association and both are described in OMIM [41, 42]. While our automated approach identified Austin-Tse et al. [41] study as the association-making study, the independent dataset we compared our result to identified Horani et al. [42] study as the source. Also, some studies used fibroblast, induced pluripotent stem cells (iPSC), tissue or organoids to make associations; however, these *in vitro* methods were not incorporated in the current version of the GPAD.

### Comparison with large language model

Large language model (LLM) like Llama-2 [43] represents the state-of-the art of NLP, harnessing vast amounts of data to understand and generate human-like text, revolutionizing the field. We test the performance of our GPAD tool against an LLM model—Llama-2. The Llama-2 model was queried with 100 randomly selected GDAs from the set of 281 mismatches identified during the validation phase with Ehrhart *et al.*’s report. The "Molecular Genetics" section of OMIM entries was isolated to serve as the input context for Llama-2, ensuring that the information provided was pertinent and within the token limits of the model.

When we compared the Llama-2 reported discovery year with our manually validated results, we have found that the Llama-2 model identifies 55% of the GDA discovery years correctly. Llama-2 correctly identified approximately 58% of the GDAs that were missed by GPAD. To test the robustness of Llama-2’s output, we conducted manual verification of a computationally randomized selection of 40 GDAs out of the 100. This verification revealed that Llama-2 correctly identified the discovery study for 50% [20] of the GDAs and accurately reported the number of studied individuals or families for 32% [16] of the cases. In case of model organism-based studies, we could not provide Llama-2 the text from “Animal Model” section of OMIM because of the model’s limitation with maximum context length. However, Llama-2 identified model organism for 37 of the 100 GDAs from the provided “Molecular Genetics” section text.

## Discussion

In this study, we present a new tool called GPAD that applies NLP to mine the rich, long-standing and manually curated OMIM database to uncover GDA-related information. The data extracted by GPAD permits tracking of trends in GDA discoveries over time and enables researchers and clinicians to quickly identify historically and contemporarily successful strategies for GDAs across all the full spectrum of MDs.

The first effort to automatically extract metadata about GDA discovery and delineation of MDs from OMIM was based on simple pattern-matching in specific sections of the narrative text [4, 24]. Therefore, information in any sentences written by the OMIM curator that did not conform to the pattern would be missed. Additionally, while most MDs with locus heterogeneity are represented in OMIM by one phenotype entry per gene, some legacy phenotype entries have not yet been split by gene, so that pattern matching will fail to accurately capture the date of GDA for many of those MDs. GPAD overcomes these shortcomings by using NLP to “read” and “understand” each sentence more naturally. GPAD also improves upon a prior effort [25] that manually curated GDA metadata, a laborious process that cannot be updated or recreated easily.

Moreover, compared to the performance shown by the generative LLM – Llama-2, designed for general chat-based question-and-answer (Q&A) tasks, GPAD identified the metadata accurately more often (Llama-2 50% compared to GPAD’s 96.16%). However, it needs further exploration to evaluate whether this performance of Llama-2 could be improved by using a domain specific LLM. Models specialized for scientific literature-based Q&A, medical knowledgebase could be candidates for such exploration. Notably, current best performing medical Q&A model Med-PaLM 2 [44] achieved 81.8% accuracy on PubMedQA dataset [45] indicating the improvements and fine-tuning these models need in this specific domain, particularly, the specialized context of OMIM along with the larger context length presents a challenge for Llama-2 and possibly similar LLMs. GPAD, by contrast, relies on deterministic and explainable methods that are less prone to such errors, enhancing its reliability. Additionally, its lightweight application size facilitates easy deployment, making it broadly accessible and usable. Furthermore, GPAD's architecture is specifically tailored for the extraction of information pertinent to GDAs, enabling it to navigate the complexities of the OMIM database effectively.

From the analysis done on the data retrieved through GPAD, we observe a recent decline in GDA discovery [24, 25]. The curators of OMIM review a large number of studies and select the ones that meet the criteria for inclusion in OMIM. This process ensures the quality and reliability of OMIM, but also requires time and resources [11, 12]. Therefore, the number of GDAs for the most recent year (2022) may be lower than expected. This number might not reflect the actual state of research, but rather the delay in cataloging which has been observed in other studies on GDAs as well [4, 24, 25].

Before the era of high throughput sequencing technologies, about ~ 150 new GDAs were described annually [4]. The advent of exome sequencing and new analysis approaches increased that rate to ~ 250 per year [24]. As these advancements clearly accelerated the pace of GDA discovery, there was a hope that we would be able to uncover much of the unknowns about rare diseases by 2020 [15, 46]. Still, we do not know the genetic origin of thousands of MDs, with many more MD anticipated discoveries [12, 24]. Our analyses show that with the decline in GDAs in recent years, we may be hampered in reaching that goal. In the last few (2020-onwards) years, we observe a decreased rate of ~ 150 discoveries per year.

Before exome sequencing, from 1998 to 2008, a reduced rate of GDAs was reported (compared to post-2008), and the trend was downward between 2005 and 2008 (Fig. 4) [47, 48]. In 2007, OMIM reports that the genetic origin of more than half of MD conditions was unknown at that time [48]. The development of exome sequencing and associated bioinformatics tools has greatly enhanced the efficiency of GDA discovery research [22, 49, 50]. After the innovation of exome sequencing, between 2011 and 2017, over 200 GDAs were discovered each year, resulting in a total of approximately 1452 associations (Fig. 4). These discoveries were further supported by studies using model organisms for confirmation.

It has been known that most MDs are rare in population [16, 51, 52]. Consistent with that knowledge, our analysis indicates that most confirmed association discoveries are made utilizing a very limited number of patients. From the evaluation based on GPAD’s result, we find that 81.57% (2704 associations) of the discoveries were made by studying less than five unrelated patients (Fig. 5A). As the majority of MD cases are rare, knowledge gained from model organism studies helped establish GDAs for more than two-thirds of cases. One noticeable trend shift in the model organism is the utilization of simpler organisms with the advent of HTS (Fig. 6). Maintaining and experimenting with higher-order organisms could be time-consuming and laborious. As HTS has become affordable and widely accessible, high-throughput experimentation favours research with simpler organisms and faster confirmation of novel GDAs. Model organisms are useful for understanding the molecular basis of novel GDAs. Although the GDA discovery rate has decreased in recent times, the number of GDAs that are validated by experimental studies using model organisms has remained relatively stable during the same period. This suggests that model organisms are still a valuable resource for verifying and exploring the functional relevance of GDAs.

The recent decline in the rate of GDA discovery after ~2016 as noted in our result can be found in previously published literature [24, 25]. Interestingly, Bamshad and colleagues explored unpublished evidence, such as discoveries made as part of the Centers for Mendelian Genomics (CMGs) [24] and suggested that the evident decline in the rate of GDA discovery is really a decline in rate of publication, and might or might not necessarily reflect a decrease in the true GDA discovery rate. Based on the analysis of GPAD’s findings, there appears to be evidence supporting the assertion. Fig. 5C illustrates a significant decline in the number of publications with single family/patient evidence, while GDAs with two or more patients has become more common during this period. We see that the number of GDAs made studying 2–5 patients were 50–75 from 2010 to 2015 and 75–100 from 2015 to 2020. However, single patient studies have decreased from 80 (2010–2015) to 40 (2015–2020). Furthermore, Fig. 4 reveals that many of the GDAs are listed as “provisional” on OMIM because of only single patient evidence. These results indicate the possibility of increased scrutiny from publishers as well as OMIM to report unconfirmed and single patient studies.

International collaborative effort to utilize case matchmaking platforms such as the MatchMatcher Exchange, members of which include GeneMatcher [53], MyGene2 [54], and DECIPHER [55], has facilitated GDA discoveries for MDs for almost a decade [56]. These platforms are connected through Matchmaker Exchange API allowing global genomic data sharing for MDs [13, 14]. It has been proposed that the lack in identifying candidate gene association can be improved by incorporating model organism related information [18] as well as variant level information (i.e. variant matching via GA4GH Application Programming Interfaces [57]).

During manual evaluation of GPAD’s performance, we have found that—often, multiple studies report novel GDA independently and concurrently. GPAD only recognizes the earliest publication in such cases and ignores the rest. Although we recognize this limitation of GPAD and we tried to address it, we could not without compromising the performance.

Our findings reflect the evolutionary trajectory of GDA discovery, from the early phenotype-focused era to the current genotype-centric methods, shaping our understanding of MDs [24]. Hence, the effectiveness and efficiency of GDAs depend on the ability to identify and interpret individual gene variants. Progress in HTS technology and bioinformatics techniques has enabled the identification of single nucleotide variants and more complex gene inactivation mechanisms, such as mobile element insertions [58] or repeat expansions [59]. It is possible that some new gene-disease associations may only be discovered once we can confidently call more complex variants present in under-studied genes that are previously not associated with any MDs [59, 60]. Nonetheless, we are still limited in our ability to identify and interpret complex structural variants and variants in non-protein coding regions [61–63]. Furthermore, the resulting phenotype could be driven by various factors, such as genetic and epigenetic modifiers [51, 64–67]. Whether these different but complicated genetic aspects uncover much of the unknown avenue of MDs and GDAs remains to be explored [51, 67–69].

## Conclusions

We have presented a novel tool—GPAD for textual analysis of OMIM for identifying GDA metadata. GPAD leverages NLP techniques to extract relevant information from OMIM such as publication information of GDAs, studied cohort and model organism. GPAD’s ability to delineate temporal trends offers researchers a macroscopic view of GDA discoveries over time, eliminating the labor-intensive task of sifting through OMIM entries. This trend analysis could aid in understanding the evolution of GDA research.

Our results and analyses of GPAD show that its method can be reliably extended to computationally extract other information from OMIM. One possible avenue of exploration could be integration of other methods of GDAs (e.g., *in vitro* methods).

Furthermore, GPAD extracted information could be used for fine -tuning LLMs and train it for variety of purpose including—broader information extraction, meaningful literature mining, surveying through current knowledge on MDs. While LLMs like Llama-2 can be powerful in extracting information, they often miss important details related to many GDAs. These language models can enhance the efficiency and accuracy of extracting complex data on MDs by understanding the contextual intricacies within OMIM’s unique content. Such a model would provide tailored insights for clinicians, researchers, and bioinformaticians, allowing for more nuanced analyses and predictions.

Finally, in this work, we have demonstrated the utility of our tool by providing the analysis on the resultant data it produces including the discovery trends. Researchers, equipped with GPAD’s data, can identify and focus on understudied areas, potentially seeking grants and collaborating with specialists in relevant fields. As research evolves, GPAD allows for the real-time tracking of new GDAs in these targeted areas, facilitating the continuous adjustment and refinement of research strategies. Ultimately, the insights offered by GPAD could be beneficial for understanding trends and developing strategies for effective and efficient disease-gene discovery.

### Supplementary Information

**Additional file 1**. Additional tables and figures for filtration criteria and dependency pattern design.

## Data Availability

The code for GPAD tool can be found at https://github.com/MTG-Lab/gpad. Installation instructions are also included. User needs to obtain an API key from OMIM (https://omim.org/api) to run the application. We are unable to publicly share the database of GDA discoveries that GPAD produced during our analysis due to licensing restrictions from OMIM. Please contact the corresponding author for access instructions.

## Abbreviations

GPAD: Gene-phenotype association discovery
OMIM: Online Mendelian Inheritance in Man
MD: Mendelian disorder
GDA: Gene-disease association
NLP: Natural language processing
NCBI: National Center for Biotechnology Information
ML: Machine learning
HTS: High throughput technologies
COVID-19: Coronavirus disease of 2019
CMG: Centers for Mendelian Genomics
POS: Parts of speech
GUI: Graphical user interface
PWA: Progressive web application
