# Supplementary material for: GPAD: a natural language processing-based application to extract the gene-disease association discovery information from OMIM
Source: BMC Bioinformatics. 2024 Feb 27;25:84. doi: 10.1186/s12859-024-05693-x (PMC10898068; doi:10.1186/s12859-024-05693-x)
Supplement: Supplementary file 1 — Additional file 1. Additional tables and figures for filtration criteria and dependency pattern design. [file 12859_2024_5693_MOESM1_ESM.pdf]

Supplementals

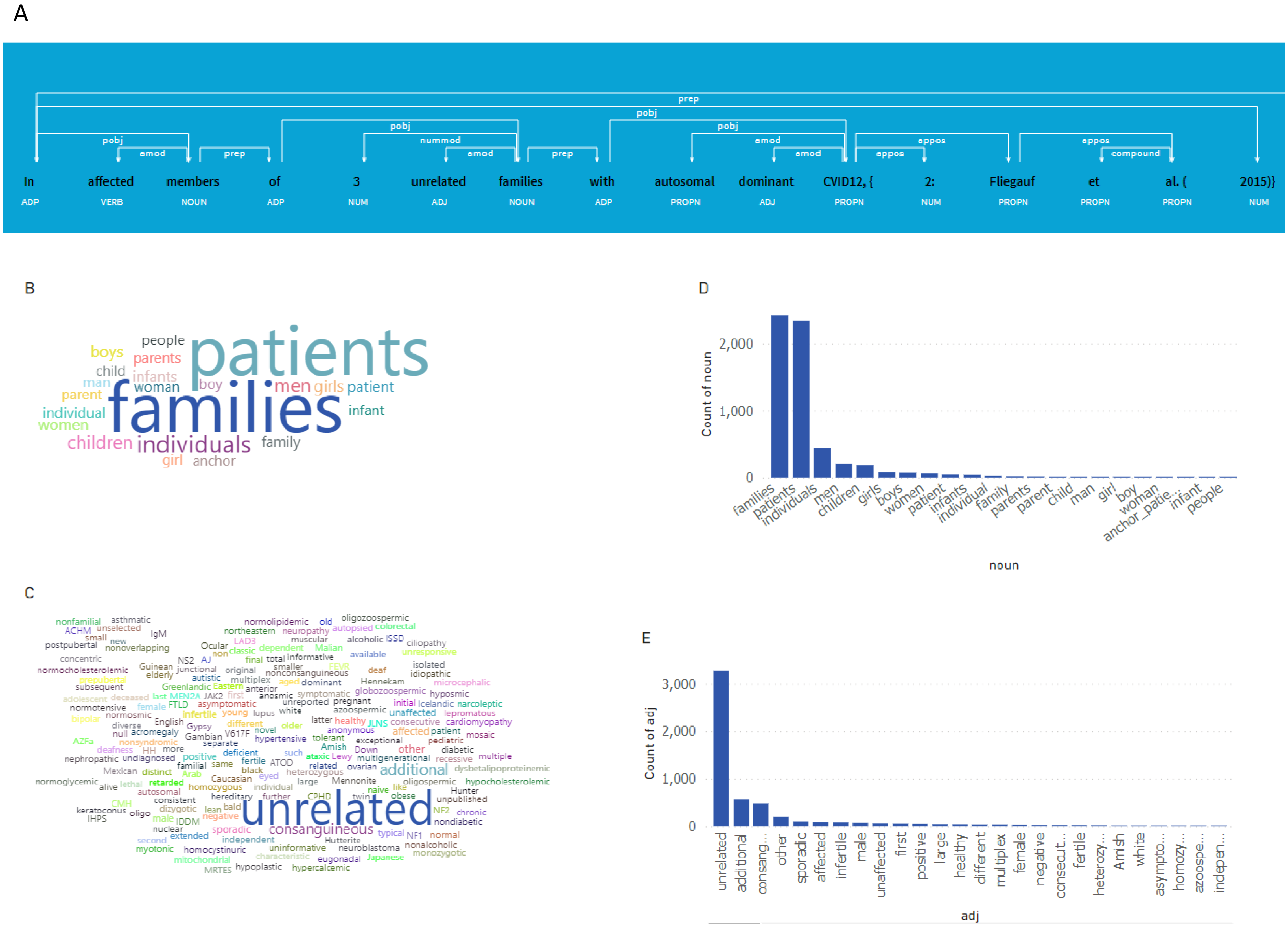

Figure S1: **(A)** Dependency diagram for a sample sentence. Here, each word is tokenized, POS-tagged and then relationship between the tags have been inferred using the SpaCy library. **(B, C)** Word cloud of identified tokens identified by individual module of the pattern and **(D, E)** shows the total count of the words for all the target texts.

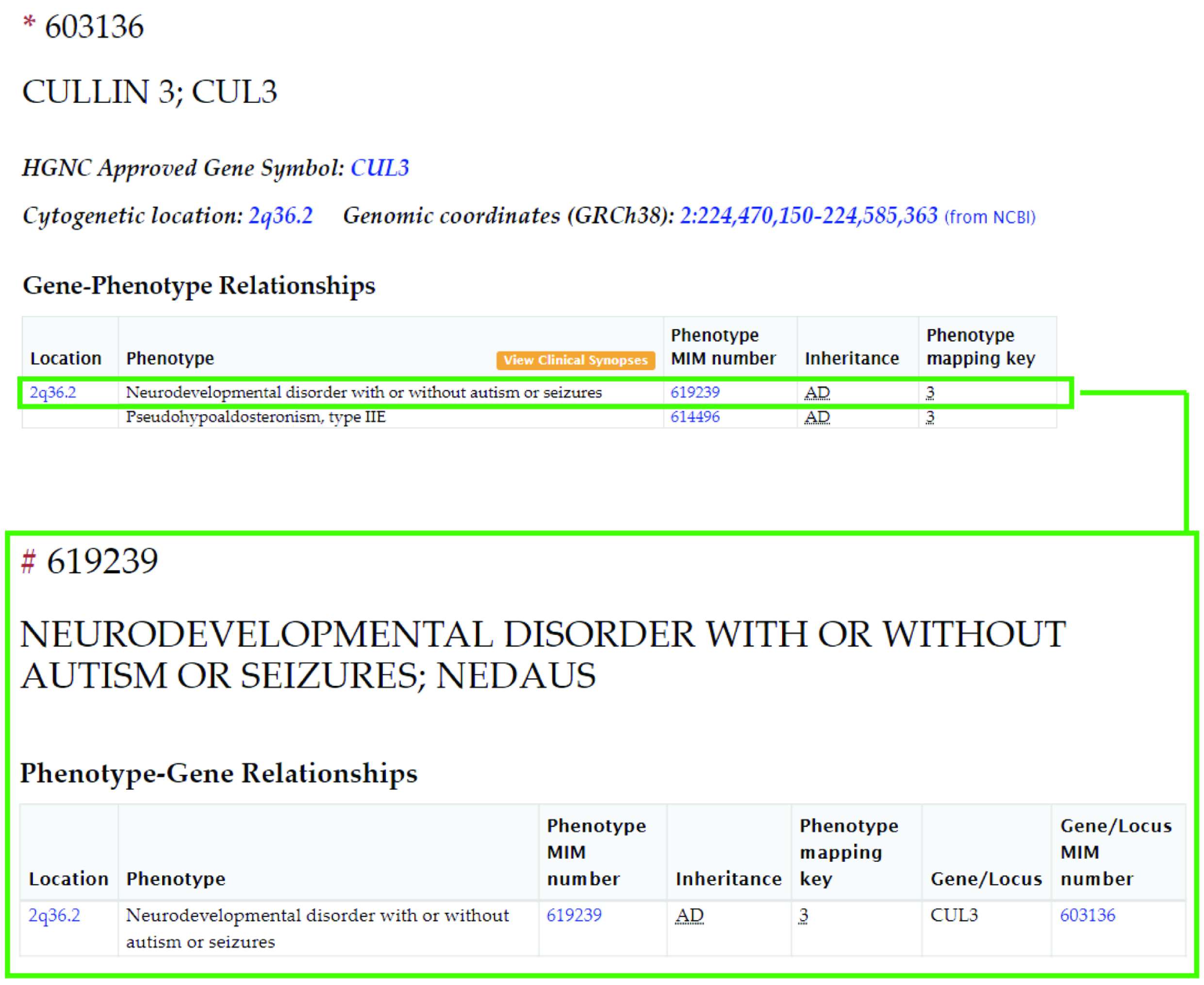

* 603136

CULLIN 3; CUL3

HGNC Approved Gene Symbol: CUL3

Cytogenetic location: 2q36.2 *Genomic coordinates (GRCh38): 2:224,470,150-224,585,363* (from NCBI)

Gene-Phenotype Relationships

Location	Phenotype	Phenotype MIM number	Inheritance	Phenotype mapping key
2q36.2	Neurodevelopmental disorder with or without autism or seizures	619239	AD	3
	Pseudohypoaldosteronism, type IIE	614496	AD	3

\# 619239

NEURODEVELOPMENTAL DISORDER WITH OR WITHOUT AUTISM OR SEIZURES; NEDAUS

Phenotype-Gene Relationships

Location	Phenotype	Phenotype MIM number	Inheritance	Phenotype mapping key	Gene/Locus	Gene/Locus MIM number
2q36.2	Neurodevelopmental disorder with or without autism or seizures	619239	AD	3	CUL3	603136

Figure S2: OMIM has two phenotypes associated with *CUL3*. GPAD uses the Phenotype-Gene relationship tables to extract the known relationship and uses the markers/indicators such as *, #, ?, {}, [] etc. to select the GDAs that are confirmed.

Anchor token 1 Anchor token 2 Selected publication Ignored publication

▼ **Molecular Genetics**

In a 3-year-old boy (family 2) with NEDAUS, Thiffault et al. (2018) identified a de novo heterozygous missense mutation in the CUL3 gene (Y58C; 603136.0008). The mutation, which was found by trio-based next-generation sequencing, was considered to be pathogenic after curation using a point-based system. Functional studies of the variant were not performed. The authors noted that several large studies had identified de novo mutations in the CUL3 gene in patients with variable neurodevelopmental disorders. For example, Kong et al. (2012) identified a de novo heterozygous loss-of-function R546X variant in 1 of 44 Icelandic individuals with autism spectrum disorder.

In a 12-year-old Brazilian girl (F1389-1) with NEDAUS, da Silva Montenegro et al. (2020) identified a de novo heterozygous nonsense mutation in the CUL3 gene (S133X; 603136.0009). The mutation, which was found by exome sequencing and confirmed by Sanger sequencing, was classified as pathogenic according to ACMG criteria.

In 3 unrelated children with NEDAUS, Nakashima et al. (2020) identified de novo heterozygous mutations in the CUL3 gene (603136.0010-603136.0012). There were 2 frameshift mutations and 1 missense mutation. The mutations, which were found by whole-exome sequencing and confirmed by Sanger sequencing, were not present in the dbSNP (build 153) or gnomAD databases. All were predicted or demonstrated to result in a loss of function and haploinsufficiency. Two patients were ascertained from a cohort of 1,230 individuals with childhood-onset epilepsy who underwent whole-exome sequencing; the third patient was identified through the GeneMatcher program.

In a 4-year-old Japanese girl with NEDAUS, Iwafuchi et al. (2021) identified a de novo heterozygous frameshift mutation in the CUL3 gene (603136.0013). The mutation, which was found by whole-exome sequencing and confirmed by Sanger sequencing, was not present in the gnomAD database. The mutation was predicted to lead to nonsense-mediated mRNA decay and haploinsufficiency. Functional studies of the variant were not performed.

▼ **Animal Model**

Dong et al. (2020) found that mice with homozygous deletion of Cul3 had reduced body size and brain weight compared with wildtype, and died prematurely. Mice heterozygous for Cul3 deletion (Cul3-deficient mice) had more than 40% reduced levels of Cul3, but were viable and fertile, survived as long as wildtype, and did not show deficits observed in mice with homozygous Cul3 deletion. Cul3-deficient mice exhibited social behavioral deficits and anxiety-like behaviors. Hippocampus of Cul3-deficient mice had increased spine density, neuronal excitability, and synaptic transmission and disrupted excitation-inhibition (E-I) balance in CA1 neurons. Similar deficits were observed in pyramidal neurons with Cul3 deficiency, demonstrating a cell-autonomous role of Cul3 for synaptic function, E-I balance, and behavior. Proteomic analysis identified Eif4g1 (600495) as a target of Cul3-dependent ubiquitination. Consequently, Cul3 deficiency increased Eif4g1 level and upregulated Cap-dependent protein synthesis in brain. Inhibition of Cap-dependent translation diminished synaptic and social deficits in Cul3-deficient mice, but it had little effect on anxiety-like behaviors. However, chemogenetic inhibition of pyramidal neuron activity in hippocampus attenuated anxiety-like behavior in mutant mice.

Figure S3: Based on the policy outlined in Figure 2C, GPAD looks for anchor token (yellow-red and orange boxes) across the input text, and select appropriate publication based on the positional proximity. This example is based on NEDAUS phenotype's "Molecular Genetics" section when looking for association evidence with *CUL3*. In this figure, GPAD choose the green-box-marked publication over the gray ones because green-boxes are in closer contextual proximity.

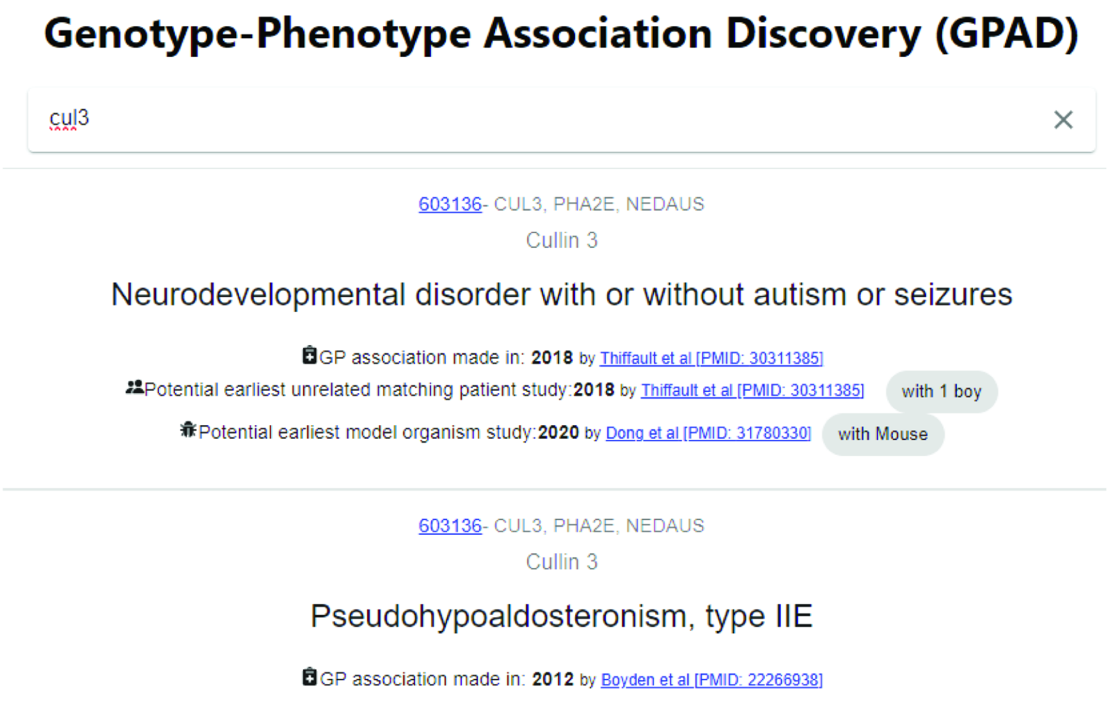

Figure S4: Screenshot showing GPAD-identified information for *CUL3*.

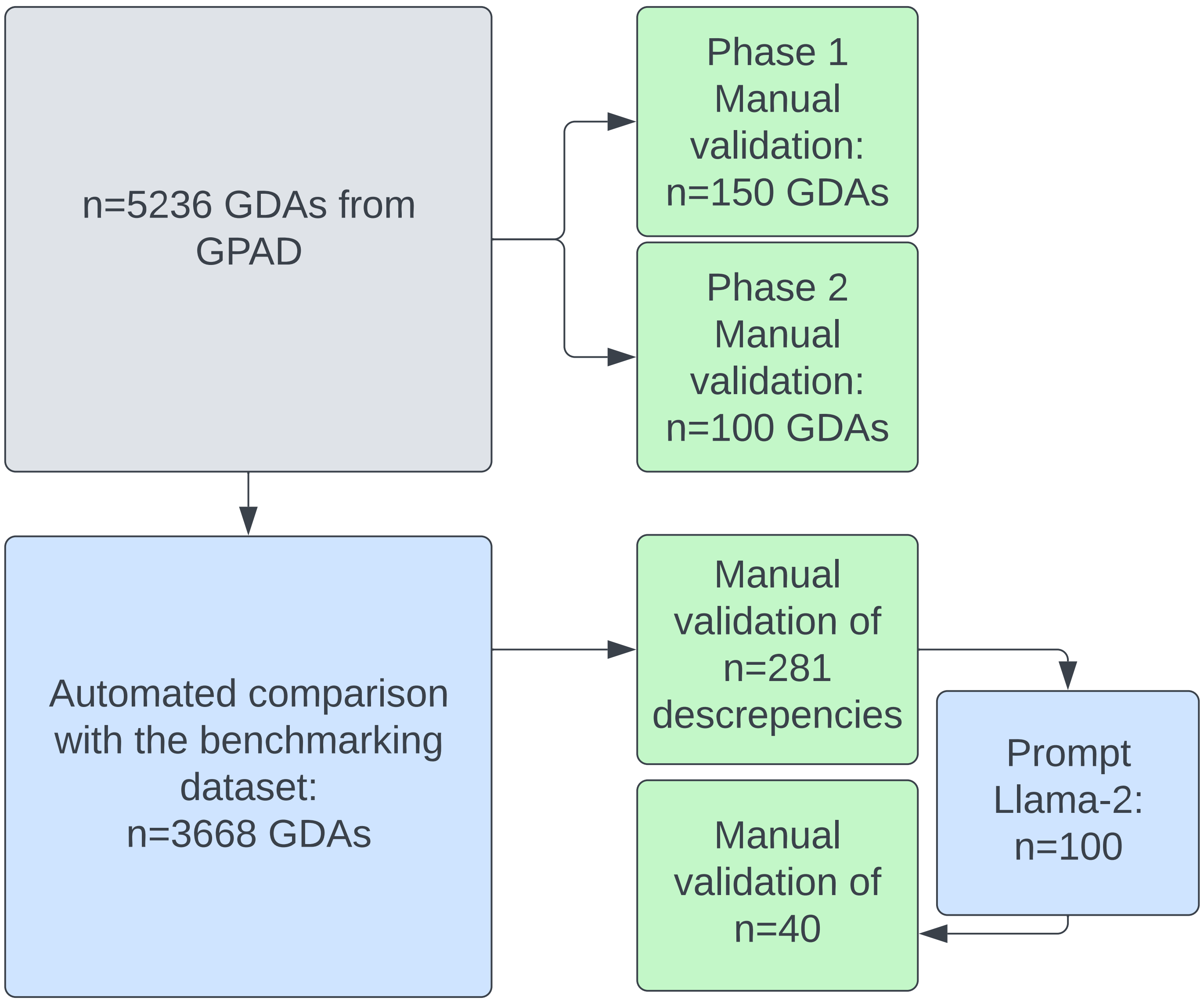

Figure S5: GPAD's performance analysis in different phases using a combination of automated/programmatic (blue boxes) and manual evaluation (green boxes).

```
You are a helpful, respectful and honest assistant. Always answer as helpfully as
possible, while being safe. Your answers should not include any harmful,
unethical, racist, sexist, toxic, dangerous, or illegal content. Please ensure
that your responses are socially unbiased and positive in nature.

If a question does not make any sense, or is not factually coherent, explain why
instead of answering something not correct. If you don't know the answer to a
question, please don't share false information.

I will give you a gene description and then a phenotype description. Then I will
ask you a question related to their association study.

<GENE_DESCRIPTION_TEXT>
<PHENOTYPE_DESCRIPTION_TEXT>

Tell me ONLY the following information of the study that associated <GENE_NAME>
(<GENE_MIM>) with <PHENOTYPE_NAME> (<PHENOTYPE_MIM>) based on the gene and
phenotype description I have provided. Provide the information in the following
format:
Year when the association was first made:
Author name of the study:
Number of case/patients that were studied:
First use of model organism (if any):
```

Figure S6: Prompt template used for Llama-2 to extract GDA related information.

Supplementary Table 1: Description of the prefix/symbols on OMIM.

Prefix/Symbol Attached with	Prefix/Symbol	Description	GPAD filtration criteria
**Phenotype mapping key**	**1**	The gene responsible for the phenotype is localized by mapping of the wildtype gene. There is an association between the gene and the phenotype, but the underlying defect is not known.	Exclude
	**2**	The phenotype is linked with the gene, but no variants have been found.	Exclude
	**3**	The gene and its molecular basis for the phenotype is known.	Include
	**4**	A phenotype that is a result of deletion or duplication of the gene(s).	Exclude
**Phenotype**	**[]**	Nondisease phenotype. These phenotypes are abnormal test values but do not qualify to mark as disiase phenotype.	Exclude
	**{}**	These mutations contributes to the susceptibility to multifactorial disorders.	Exclude
	**?**	Indicates GDAs that are provisional.	Exclude

Supplementary Table 2: Specification to run Llama-2 on selected OMIM text.

Parameter	Value/Version
**Model version**	Llama-2-7B-Chat
**GPU Memory**	64 GB (V100 GPU)
**--nproc_per_node**	1
**--max_seq_len**	4096
**--max_batch_size**	2
**Python**	v3.11
**CUDA**	11.7
